# Luminescence-based *in vivo* monitoring of NF-κB activity through a gene delivery approach

**DOI:** 10.1186/1478-811X-11-19

**Published:** 2013-03-21

**Authors:** Fernando G Osorio, Jorge de la Rosa, José MP Freije

**Affiliations:** 1 Departamento de Bioquímica y Biología Molecular, Facultad de Medicina, Instituto Universitario de Oncología, Universidad de Oviedo, Oviedo, 33006, Spain

## Abstract

**Background:**

Monitoring activity of specific signaling pathways *in vivo* is challenging and requires highly sensitive methods to detect dynamic perturbations in whole organisms.

**Results:**

*In vivo* gene delivery of a luciferase reporter followed by bioluminiscence imaging allows measuring NF-κB activity in mice liver and lungs.

**Conclusions:**

This protocol allows a direct measure of NF-κB activity through quantification of bioluminescence signal, demonstrating its accuracy and sensitivity in different animal models and experimental conditions. Variants could be also applied for the analysis of NF-κB activity in different tissues or for studying other signaling pathways *in vivo*.

## Background

Monitoring biological processes *in vivo* remains experimentally challenging. Visualization of biochemical perturbations in whole organisms requires highly sensitive methods that allow detection in a quantitative and reproducible manner [1-3]. Reporter gene approaches are based on the use of specific regulatory sequences attached to genes that confer organisms a property that could be easily detected and quantified, typically light or fluorescence emission [4]. Different luciferase and fluorescent proteins-based strategies have been developed for monitoring signaling pathways involved in cancer and aging [5-11].

Most of the available strategies rely on the generation of transgenic mice that stably express reporter genes. These mutant animals allow dynamic studies of complex processes, as the activity of the monitored signaling pathways could be recorded at different time points during lifetime. The main limitation of these approaches is that they imply the generation of transgenic strains, involving a long and expensive process that requires the use of a large number of experimentation animals. An alternative approach is described herein, based on the use of *in vivo* gene delivery strategies that potentially overcome previous limitations, allowing a fast and reliable quantification of the activity of signaling pathways in a wide variety of experimental models and physiological situations. Here we specifically describe a luciferase-based approach to measure *in vivo* NF-κB activity in different mice tissues.

NF-κB is involved in the response against a large variety of external and internal stress signals, having essential roles in inflammation, immune response, cell proliferation and protection against apoptosis [12-14]. Consequently with its key role in cellular stress response, NF-κB aberrant activation has been linked with several pathophysiological conditions including cancer and accelerated aging [15,16]. In this regard, it has been described that not only acute NF-κB activation is related with disease but also subtle changes in NF-κB activity that persist in time have important biological consequences, giving rise to chronic inflammatory conditions. The monitoring of these subtle changes in NF-κB activity has been especially challenging.

### NF-κB luciferase reporter-based assay

NF-κB activation is most often monitored in cultured cells using a non-viral vector system that encodes firefly luciferase reporter gene under the control of a minimal cytomegalovirus (CMV) promoter and tandem repeats of the NF-κB transcriptional response element. The reporter gene will be only transcribed when NF-κB signaling pathway is active; otherwise Rel transcription factors are sequestered in the cytoplasm by an inhibitory complex. Inflammatory signals trigger NF-κB activation that involves nuclear translocation of the NF-κB transcription factors. The reporter gene will be transcribed, like endogenous NF-κB targets, proportionally to the magnitude of NF-κB activation and the time persistence of the induction signal. Monitoring bioluminescence emission by the tissues upon luciferin administration allows visualization of NF-κB activity *in vivo*.

### In vivo gene delivery

Reporter-based strategies *in vivo* require using highly efficient methods of gene delivery that ensure the transfection of a high proportion of cells. For liver gene delivery, we have selected hydrodynamic transfection, a simple and efficient non-viral method that allows transfection of a naked DNA in a high proportion of the liver hepatocytes [17]. Rapid injection of a high-volume (10%, vol/weight) solution of the naked plasmid DNA through the tail vein generates a hydrodynamic pressure that expands liver endothelium resulting in DNA uptake by the hepatocytes [18,19]. Although the protocol is optimized for mice tissues, the technique could be modified for other animal models, even muscles of larger animals [20].

For lung delivery we have used DNA complexed with a polycation, polyethyleneimine (PEI). DNA-PEI complexes are efficiently internalized into the cells and the DNA is subsequently transported into the nucleus [21,22]. Systemic administration of DNA complexes results in gene delivery primarily to the lungs, reaching all the anatomical areas of this organ; this is an important advantage of this method as compared with others based on instillation, where only the proximal area is achieved [23].

## Methods

### Reagents

•Mice. We routinely use 6–20 week-old C57BL/6 mice, but we have also successfully used mice from other strains (129S1/SvImJ, BALB/c and CD1) as well as several genetically modified mouse models. All the animal experiments were performed with the aproval of the Committee for Animal Experimentation of the Universidad de Oviedo.

•NF-κB luciferase gene reporter (Qiagen, cat. no. CCS-013 L).

•In vivo jet-PEI™. Linear 22-kDa polyethylenimine (see reagent setup) (Polyplus transfection, cat. no. 201-10G).

•Autoclaved Ringer’s solution (123 mM NaCl, 5 mM KCl and 1.5 mM CaCl_2_).

•Autoclaved saline. 0.9% (wt/vol) sodium chloride.

•Anesthetic. Isoflurane (Abbott laboratories, cat. no. 571329.8).

•D-Luciferin firefly luciferase substrate, potassium salt (Melford laboratories, cat. no. L1350).

•*Escherichia coli* lipopolysaccharide (LPS) (Sigma, L2630).

•Sodium salicylate (Sigma, S3007).

•Endo-Free plasmid maxi kit (Qiagen, cat. no. 12362).

### Equipment

•Xenogen or other *in vivo* luminescence imaging system (we have used Xenogen IVIS 100 series for the recording and data analysis).

•Isoflurane vaporizer.

•27-G needle (BD microlance, cat. no. 300635).

•2 mL. syringe (BD discardit, cat. no. 300928).

•45–50°C waterbath and/or heat lamp.

•Shaver to remove mouse hair.

•Scale for weighing mice.

### Reagent setup

#### Preparation of NF-κB luciferase reporter gene

Plasmids are purified by ion-exchange chromatography using EndoFree plasmid maxi kit following the manufacturer’s instructions. It is very important that plasmids used for *in vivo* gene delivery be free of endotoxins.

#### Luciferin stock solution

Prepare a stock solution of luciferin (14.3 mg/mL) by dissolving 1 g substrate in 70 mL saline solution (0.9% sodium chloride). Filter the solution through a 0.22 μm sterile filter. Store reconstituted substrate in aliquots at −80°C. Use 0.2 mL per mice for intraperitoneal injection.

#### Preparation of PEI/DNA complexes

For an effective cell delivery of plasmid DNA, the overall ionic charge of the DNA complexes needs to be cationic. The amount of nitrogen (N) in the PEI and phosphate in the DNA (P) determines the charge of the complexes. In practice, the best transfection results are obtained with a N/P ratio of 5–10. For this specific purpose we have successfully used an N/P ratio of 8 and 50 μg of DNA per mice, using a total amount of 8 μL of jetPEI™ reagent. If using other mouse strains or targeting a different organ, a dose response should be carried out to determine the optimal PEI-DNA ratio. Calculate the amount of *in vivo*-jetPEI™ in each case as follows:

$$\mathrm{\mu L}\;\text{of}\;\text{in}\;\text{vivo}-\text{jetPE}I^{\text{TM}}=\frac{\left(\mathrm{\mu g}\;\mathrm{of}\;\text{DNA}\times 3\right)\times N/P\;\text{ratio}}{150}$$

### Equipment setup

#### General setup considerations

Prepare all the necessary material, warm up the water bath, turn on the vaporizer anesthesia machine, and switch on the heat lamp, placing a cage under it for mice recovery after the procedure.

### Procedure

#### Mice preparation (Timing 5 min)

1) Check the vaporizer system to ensure adequate amounts of supply gas (O_2)_ and isoflurane for duration of the procedure. Make sure system is set to flow to induction chamber. Turn on supply gas. Weigh mouse before this point.

2) Turn on flowmeter between 500–1000 mL/min and place animal in the induction chamber. Turn on vaporizer to 2-3%. Monitor animals until recumbent.

3) Switch system to flow to nosecone, remove animal from induction chamber and place in nosecone. Turn the vaporizer to 1.5-2%.

### Preparation of DNA sample and hydrodynamic injection (Timing 15 min)

4) Calculate the volume required for hydrodynamic injection based on the weight of the mouse (10%, vol/weight). For example: a 15 g mouse will be injected with 1.5 mL of DNA solution. We normally use a 10 μg/mL DNA solution although this amount could be varied depending on the experimental requirements.

5) Dilute DNA in Ringer’s solution and set a syringe according with the calculated volume.

6) Place the tail of the anesthetized mice in 45–50°C heated water for 30 s. This will make tail veins more easily visualized.

7) Mice have two lateral tail veins, both of which are superficial and could be used for the procedure. Place the tail with the lateral side up between your fingers. Inject the needle into the vein 2–3 cm from the tail tip. You can often see the needle into the vein.

8) Press with force the plunger of the syringe, trying to inject the entire volume in less than 10 s. When finished, remove the needle and apply light pressure to the injection site until the bleeding stops.

9) In general, a cohort of several mice could be injected one after the other, and up to 15–20 mice could be easily injected in a session.

10) The recovery phase could last up to an hour for full recovery; we recommend placing the injected mice under a heat lamp to avoid hypothermia.

### Preparation of PEI/DNA complexes and injection into mice (Timing 15 min)

11) Dilute 50 μg of the plasmid in 100 μL of 5% glucose solution. Vortex gently. Dilute 8 μL of *in vivo*-jetPEI™ reagent in 100 μL of 5% glucose solution. Combined both and incubate the 200 μL final volume 15 min at room temperature (RT). From this point the complexes are stable for 2 hours at RT. Critical step, proceed immediately to the next point.

12) Proceed from step 3 and place the tail of the anesthetized mice under 45–50°C heated water for 30 s. Inject 0.2 mL PEI-complexed DNA into the lateral vein.

13) Place mice under the heat lamp for recovery.

### In vivo bioluminescence imaging (Timing 30 min)

14) Anesthetize mice using isoflurane vaporizer coupled to the IVIS imaging system. Shave the ventral (for imaging of liver) or dorsal (for imaging of lungs) area of the mice. This is especially important for an adequate imaging of dark-coated mice such as C57BL/6.

15) Inject 0.2 mL luciferin i.p. and wait for 5 min. Imaging should be carried out between 5 and 30 min after luciferin injection. We usually perform imaging of several mice at the same time.

16) Image the mice according with the equipment instructions; we have good results with the Xenogen’s IVIS Living image program selecting a medium binning and a variable exposure time depending on signal intensity. Avoid signal saturation in order to obtain a lineal signal.

### Data analysis

**Figure 1:**
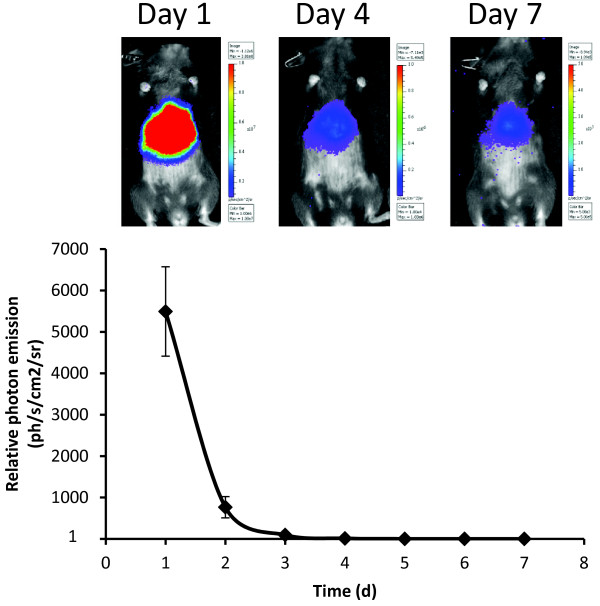
**Representative images of the bioluminescence signal registered in mice at three different time points after hydrodynamic transfection (1, 4 and 7 days).** Plot represents bioluminescence signal quantification of C57BL/6 complete mice cohort (n=10) during a week after injection. Mean values are represented as relative values of photon flux per second and square centimeter, error bars indicate SEM.

17) Change image setting from counts to photons. Photons are absolute physical units that measure the light emission from the subject. The radiance unit of photons/sec/cm2/sr is the number of photons per second that leave a square centimeter of tissue and radiate into a solid angle of one steradian (sr). Measurements in units of radiance automatically take into account camera settings and allow comparing data obtained from different animals or from the same animal in different days.

18) For each mouse draw a measurement region of interest (ROI), and adjust the threshold value depending on the intensity of the signal. Obtain a background-corrected ROI measurement by subtracting an average background ROI from the measured ROI by using software computation options.

19) Critical step. We recommend measuring basal NF-κB activity starting at 72 hours after the plasmid injection, the reason is that hydrodynamic delivery procedure could induce a transient inflammatory response in the liver that interfere with the measurements (Figure 1). We have not seen this effect using PEI/DNA complexes.

## Results and discussion

A cohort of C57BL/6 mice was hydrodynamically transfected with the NF-κB luciferase reporter (Figure 1). Bioluminescence signals were recorded each day during a week. According to the data shown, bioluminescence signal is especially high during the first 48 hours after the injection, rapidly diminishing thereafter and reaching stable values 72 hours after the injection. This phenomenon is due to a transient inflammatory response caused by the hydrodynamic procedure; for this reason, we recommend measuring NF-κB activity at least 72 hours after injection in order to prevent potential interference in the signal recorded.

**Figure 2:**
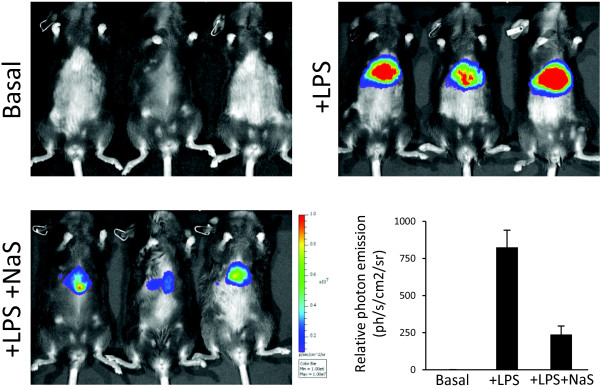
**Constitutive and LPS-induced measure of liver NF-κB activity.** Representative pictures are shown of three C57BL/6 mice before LPS administration and 24 hours after the injection, half of the cohort also received an injection of sodium salicylate (NaS). Plot represents relative mean bioluminescence values as photon flux per second and square centimeter, error bars indicate SEM.

A potential application of this method to study basal and acute induced activity of NF-κB in mice livers is described in the Figure 2. To this end, we first measured NF-κB basal activity in C57BL/6 mice and then we induced acute inflammation with *E. coli* lipopolysaccharide (LPS). Additionally, half of the mice injected with the LPS received a dose (200 mg/Kg) of sodium salicylate, a powerful inhibitor of NF-κB signaling. Quantification of the bioluminescence signals demonstrates the sensitivity and reproducibility of the method described.

**Figure 3:**
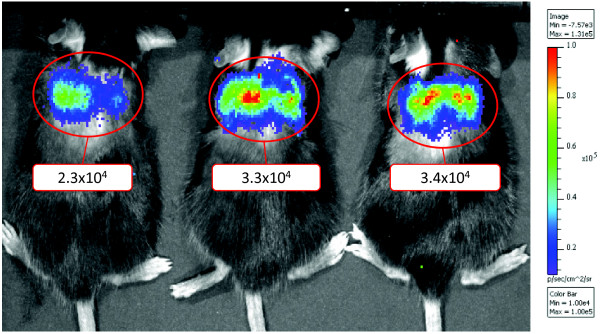
**NF-κB basal activity in mouse lungs.** Representative image of three C57BL/6 mice 24 hours after the injection of the PEI/DNA complexes.

The analysis of constitutive NF-κB activity in the lungs is showed in Figure 3. PEI/DNA complexes allow an efficient and highly-specific delivery of the NF-κB reporter to the lungs.

The method described herein has been successfully used for studying constitutive and acute-induced NF-κB signaling in mice tissues [12,24]. The use of this approach allowed us to demonstrate chronic activation of NF-κB in two different mouse models of accelerated aging and to monitor the response in these models to non-steroid anti-inflammatory drugs [12,16]. These results support the utility of this approach to study both chronic conditions as well as acute inflammatory responses.

## Conclusions

The methodology described herein includes a step-by-step procedure for liver and lung gene delivery, recording of the bioluminescence signal and data analysis. Besides, we provide experimental validation of the protocol, demonstrating the sensitivity and reproducibility of the method.

The possibility of using viral vectors with different tissue tropisms for the reporter delivery potentially allows monitoring NF-κB in any tissue. Additionally, the same strategy may be applied for studying different signaling pathways, replacing the NF-κB response element with other regulatory sequences in the luciferase reporter system.

## Competing interests

The authors declare that they have no competing interests.

## Authors’ contributions

JMPF designed the overall protocol and supervised the project. FGO and JR carried out the experiments. FGO and JMPF wrote the manuscript. All authors read and approved the final manuscript.
